# Supplementary material for: Mutational alterations in the QRDR regions associated with fluoroquinolone resistance in Pseudomonas aeruginosa of clinical origin from Savar, Dhaka
Source: PLoS One. 2025 Feb 24;20(2):e0302352. doi: 10.1371/journal.pone.0302352 (PMC12002805; doi:10.1371/journal.pone.0302352)
Supplement: S2 File — (DOCX) [file pone.0302352.s002.docx]

**Supplementary information**

**Mutational alterations in the QRDR regions associated with fluoroquinolone resistance in *Pseudomonas aeruginosa* of clinical origin from Savar, Dhaka**

Md. Shamsul Arefin^1^, Meftahul Jannat Mitu^1^, Shomaia Yasmin Mitu, Azmeri Nurjahan^2^, Mir Mobin^1^, Shamsun Nahar, Hasnain Anjum^1,3^, M. Hasibur Rahman^1^

^1^ Department of Microbiology, Jahangirnagar University, Savar, Dhaka.

^2^ Department of Biotechnology and Genetic Engineering, Jahangirnagar University, Savar, Dhaka

^3^ Department of Microbiology, Primeasia University, Banani, Dhaka.

Dear editor,

Please find attached Supplementary Table 1 and 2 describing Logistic Regression Model of Predicting Resistance in *Pseudomonas aeruginosa* in the different clinical specimens. This data has been previously published by us (Anjum *et al.,* 2023), but is now included as supplementary data as per the suggestion of Reviewer #3. I hope you’ll find this useful.

Sincerely,

Md. Hasibur Rahman

**Supplementary Table 1.** Collection of samples and culture results

| Sample type | Samples collected from EMCH | | Samples collected from GMCH | | Total Samples | Proportion of Positive Samples |
| --- | --- | --- | --- | --- | --- | --- |
|  | Number of Samples | Positive for *P. aeruginosa* | Number of Samples | Positive for *P. aeruginosa* |  |  |
| Urine | 72 | 16 | 43 | 7 | 115 | 23 (20%) |
| Urinary Catheter | 7 | 4 | 5 | 1 | 12 | 5 (42%) |
| Sec. wound infection | 12 | 7 | 9 | 4 | 21 | 11 (52%) |
| Pus | 37 | 5 | 32 | 5 | 69 | 10 (14%) |
| Burn wound | 8 | 2 | 2 | 0 | 10 | 2 (20%) |
| Blood | 9 | 1 | 0 | 0 | 9 | 1 (11%) |
| Tracheal aspirate | 2 | 1 | 0 | 0 | 2 | 1 (50%) |
| Total | 147 | 36 | 91 | 17 | 238 | 53 (22%) |

Here, GMCH = Gonoshasthaya Samaj Vittik Medical College & Hospital; EMCH = Enam Medical College & Hospital.

**Supplementary Table 2.** Logistic Regression Model of Predicting Resistance in *Pseudomonas aeruginosa* in the different clinical specimens.

|  | **Susceptible** | **Resistance** | **Resistance** | |
| --- | --- | --- | --- | --- |
| **Specimens** |  |  | **OR (95% CI)** | **p-value** |
| **AMP** |  |  |  |  |
| Urine | 0 | 23(100%) | - |  |
| Pus | 0 | 10(100%) |  |  |
| Secondary wound infection swab | 0 | 11(100%) | - |  |
| Catheter swab | 0 | 5(100%) |  |  |
| Others^$^ | 0 | 9(100%) | - |  |
| **CAZ** |  |  |  |  |
| Urine | 23(100%) | 0 | Ref. (1) |  |
| Pus | 10(100%) | 0 | - |  |
| Secondary wound infection swab | 8(72.7%) | 3(27.3%) | 0.79(0.68, 3.85) | 0.882 |
| Catheter swab | 3(60.0%) | 2(40.0%) | 1.65(0.78, 4.25) | 0.525 |
| Others^$^ | 5(55.6%) | 4(44.4%) | 0.99(0.35, 4.00) | 0.999 |
| **CRO** |  |  |  |  |
| Urine | 0 | 23(100%) | - |  |
| Pus | 0 | 10(100%) | - |  |
| Secondary wound infection swab | 0 | 11(100%) |  |  |
| Catheter swab | 0 | 5(100%) | - |  |
| Others^$^ | 0 | 9(100%) | - |  |
| **CTX** |  |  |  |  |
| Urine | 0 | 23(100%) | Ref. (1) |  |
| Pus | 1 | 9(90.0%) | 0.98(0.88, 3.28) | 0.788 |
| Secondary wound infection swab | 0 | 11(100%) | - |  |
| Catheter swab | 1(20.0%) | 4(80.0%) | 3.01(1.88, 5.04) | 0.032 |
| Others^$^ | 1(11.1%) | 8(88.9%) | 0.99(0.17, 7.66) | 0.887 |
| **IMP** |  |  |  |  |
| Urine | 19(82.6%) | 4(12.1%) | Ref. (1) |  |
| Pus | 10(100%) | 0 | - |  |
| Secondary wound infection swab | 6(54.6%) | 5(45.5%) | 3.97(0.79, 19.7) | 0.093 |
| Catheter swab | 1(20.0%) | 4(80.0%) | 18.9(1.65, 29.4) | 0.018 |
| Others^$^ | 1(25.0%) | 3(75.0%) | 14.3(1.16, 23.6) | 0.038 |
| **CIP** |  |  |  |  |
| Urine | 19(82.6%) | 4(17.4%) | Ref. (1) |  |
| Pus | 10(100.0%) | 0 | - |  |
| Secondary wound infection swab | 7(63.6%) | 4(36.4%) | 2.69(0.53, 13.9) | 0.231 |
| Catheter swab | 1(20.0%) | 4(80.0%) | 18.9(1.65, 29.4) | 0.018 |
| Others^$^ | 1(25.0%) | 3(75.0%) | 14.3(1.16, 23.6) | 0.038 |

|  | **Susceptible** | **Resistance** | **Resistance** | |
| --- | --- | --- | --- | --- |
| **Specimens** |  |  | **OR (95% CI)** | **p-value** |
| **LEV** |  |  |  |  |
| Urine | 18(78.3%) | 5(21.7%) | Ref. (1) |  |
| Pus | 10(100%) | 0 | - |  |
| Secondary wound infection swab | 7(63.6%) | 4(36.4%) | 2.05(0.42, 9.97) | 0.370 |
| Catheter swab | 1(20.0%) | 4(80.0%) | 14.4(1.30, 21.5) | 0.018 |
| Others^$^ | 1(25.0%) | 3(75.0%) | 10.8(1.09, 17.3) | 0.049 |
| **CN** |  |  |  |  |
| Urine | 20(87.0%) | 3(13.0%) | Ref. (1) |  |
| Pus | 10(100%) | 0 | - |  |
| Secondary wound infection swab | 7(63.6%) | 4(36.4%) | 2.45(0.42, 9.97) | 0.570 |
| Catheter swab | 1(20.0%) | 4(80.0%) | 24.5(3.32, 18.2) | 0.004 |
| Others^$^ | 1(25.0%) | 3(75.0%) | 20.1(2.34, 19.9) | 0.009 |
| **AZM** |  |  |  |  |
| Urine | 4(17.4%) | 19(82.6%) | Ref. (1) |  |
| Pus | 4(40.0%) | 6(60.0%) | 3.16(0.60, 16.6) | 0.174 |
| Secondary wound infection swab | 4(36.4%) | 7(63.6%) | 2.69(0.53, 13.9) | 0.231 |
| Catheter swab | 1(20.0%) | 4(80.0%) | 1.19(0.10, 13.6) | 0.890 |
| Others^$^ | 2(50.0%) | 2(50.0%) | 5.26(0.51, 16.3) | 0.172 |
| **TET** |  |  |  |  |
| Urine | 2(8.70%) | 21(91.3%) | Ref. (1) |  |
| Pus | 1(10.0%) | 9(90.0%) | 1.16(0.09, 14.6) | 0.905 |
| Secondary wound infection swab | 2(18.2%) | 9(81.8%) | 2.23(0.32, 15.5) | 0.419 |
| Catheter swab | 1(20.0%) | 4(80.0%) | 2.51(0.21, 20.3) | 0.471 |
| Others^$^ | 0 | 4(100%) | 4.48(0.97, 14.4) | 0.078 |

Others^$^ Tracheal aspirate, blood and burn wound.

Logistic regression model was used to estimate the p-value. OR= Odds Ratio; CI= Confidence Interval

AMP=Ampicillin; CRO=Ceftriaxone; CTX=Cefotaxime; CAZ=Ceftazidime; IPM= Imipenem; CIP=Ciprofloxacin; LEV=Levofloxacin; CN=Gentamycin; TET=Tetracycline; AZM=Azithromycin

**Supplementary Table 3.** Risk of MDR based on specimens

|  | Non-multidrug resistant | Multidrug resistant (MDR) | Multidrug resistant (MDR) | |
| --- | --- | --- | --- | --- |
| Specimens |  |  | OR(95% CI) | p-value |
| Urine | 6(18.2%) | 27(81.8%) | Ref. (1) |  |
| Secondary wound infection swab | 1(9.09%) | 10(90.9%) | 2.23(0.05, 4.22) | 0.065 |
| Catheter swab | 0 | 5(100%) | 2.52(1.01, 11.9) | 0.049 |
| Others^$^ | 1(25.0%) | 3(75.0%) | 1.51(0.13, 17.1) | 0.244 |

Others^$^ Tracheal aspirate, blood and burn wound.

Logistic regression model was used to estimate the p-value. Odds Ratio; CI= Confidence Interval
