# Supplementary material for: Mutational alterations in the QRDR regions associated with fluoroquinolone resistance in Pseudomonas aeruginosa of clinical origin from Savar, Dhaka
Source: PLoS One. 2025 Feb 24;20(2):e0302352. doi: 10.1371/journal.pone.0302352 (PMC12002805; doi:10.1371/journal.pone.0302352)
Supplement: S1_Raw_Images — (DOCX) [file pone.0302352.s003.docx]

**Supplementary images**

**Mutational alterations in the QRDR regions associated with fluoroquinolone resistance in *Pseudomonas aeruginosa* of clinical origin from Savar, Dhaka**

Md. Shamsul Arefin^1^, Meftahul Jannat Mitu^1^, Shomaia Yasmin Mitu, Azmeri Noorjahan^2^, Mir Mobin^1^, Shamsun Nahar, Hasnain Anjum^1,3^, M. Hasibur Rahman^1^

^1^ Department of Microbiology, Jahangirnagar University, Savar, Dhaka.

^2^ Department of Biotechnology and Genetic Engineering, Jahangirnagar University, Savar, Dhaka

^3^ Department of Microbiology, Primeasia University, Banani, Dhaka.

Dear editor,

Please find attached the original gel images used in this study herewith as per request. The images have been annotated according to instructions. Additional images from phenotypic tests and gel electrophoresis have been included in this file as well, as per the comment from Reviewer 1. I hope you’ll find this useful.

Sincerely,

Md. Hasibur Rahman

**Original Gel Images:**

X	X	X	X	X	X	X	X	X	X	X	X	X	X	X	X

X	X	X	X	X	X	X	X	X	X	X	X

500bp

400bp

400bp

428bp

500bp

17	18	19	20	21	22	23	24	25	26	27	28	29	30	31	32

1	2	3	4	5	6	7	8	9	10	11	12	13	14	15	16

Original gel 1: Electrophoresis showing PCR amplicons for PMQR gene *qnrS* on 1% agarose gel stained with ethidium bromide: Lane 1 and 17 represents 100bp DNA marker (New Englad Biolabs, USA); lane 2 represents positive control showing 428 bp amplicon of *qnrS* gene; lane 3 shows Negative control; lane 4-16 and 18-32 displays test isolates showing negative amplification of *qnrS.* Images were taken using a UV transilluminator at 312nm wavelength following destaining. The third panel of Fig 1 is taken from this gel.

X	X	X	X	X	X	X

X	X	X	X	X	X	X

1	2	3	4	5	6	7	8	9	10	11

600bp

500bp

580bp

500bp

400bp

300bp

390bp

12	13	14	15	16	17	18	19	20	21	22

Original gel 2: Electrophoresis showing PCR amplicons for PMQR genes *qnrB* (top row) and *qnrA* (bottom row) on 1% agarose gel stained with ethidium bromide: Lane 1 and 12 represents 100bp DNA marker (New Englad Biolabs, USA); lane 2 and 13 represents positive controls showing 390 bp and 580 bp amplicons of *qnrB* and *qnrA* gene, rspectively; lane 3 and 14 shows Negative control; lane 4-11 and 15-22 displays test isolates showing negative amplification of *qnrB* (top row) and *qnrA* (bottom row)*.* Images were taken using a UV transilluminator at 312nm wavelength following destaining. The first and second panel of Fig 1 is taken from this gel (bottom and top row, respectively).

**Supplementary Images:**

Supplementary Fig 1: *Pseudomonas aeruginosa* on Cetrimide Agar showing a. green pigmented colonies and b. showing fluorescence under ultraviolet light.

Supplementary Fig 2: Cultural and biochemical characterization of *Pseudomonas aeruginosa*

Supplementary Fig 3: Agarose gel electrophoresis of 16S rDNA of representative isolates; lane 1 represents 1kb marker and from lane 2 to lane 9 represents the amplified product of 16S rDNA.

Supplementary Fig 4: Antibiogram test using disk diffusion method.

Supplementary Fig 5: Assessment of antimicrobial resistance using Minimum Inhibitory Concentration (MIC) assay.

Supplementary Fig 6: Agarose Gel Electrophoresis showing PCR amplicons for *gyrA* gene**.** Lane 1 shows 100 bp marker DNA ladder. Lane 2-11 represents 416 bp PCR amplicons for *gyrA* gene.

Supplementary Fig 7: Agarose Gel Electrophoresis showing PCR amplicons for *parC* gene. Lane 1 shows 100bp marker DNA ladder. Lane 2-9 represents 303 bp PCR amplicons for *parC* gene.
