# Supplementary material for: Mutational alterations in the QRDR regions associated with fluoroquinolone resistance in Pseudomonas aeruginosa of clinical origin from Savar, Dhaka
Source: PLoS One. 2025 Feb 24;20(2):e0302352. doi: 10.1371/journal.pone.0302352 (PMC12002805; doi:10.1371/journal.pone.0302352)
Supplement: Attachment — Submitted filename: Response to Reviewers.docx [file pone.0302352.s004.docx]

**Mutational alterations in the QRDR regions associated with fluoroquinolone resistance in Pseudomonas aeruginosa of clinical origin from Savar, Dhaka**

Journal requirements:

When submitting your revision, we need you to address these additional requirements.

1. Please ensure that your manuscript meets PLOS ONE's style requirements, including those for file naming. The PLOS ONE style templates can be found at

<https://journals.plos.org/plosone/s/file?id=wjVg/PLOSOne_formatting_sample_main_body.pdf> and

<https://journals.plos.org/plosone/s/file?id=ba62/PLOSOne_formatting_sample_title_authors_affiliations.pdf>

**Author’s reply:** Thank you so much. The file and author affiliation has been updated according to the style template.

2. Please amend the manuscript submission data (via Edit Submission) to include author Azmeri Noorjahan.

**Author’s reply:** Thank you so much. The author’s name has been included.

3. Please amend your authorship list in your manuscript file to include authors Azmery Nurjahan, Shamsun Nahar and Shomia Yasmin Mitu.

**Author’s reply:** Thank you so much. The authorship list has been updated accordingly.

4. PLOS ONE now requires that authors provide the original uncropped and unadjusted images underlying all blot or gel results reported in a submission’s figures or Supporting Information files. This policy and the journal’s other requirements for blot/gel reporting and figure preparation are described in detail at <https://journals.plos.org/plosone/s/figures#loc-blot-and-gel-reporting-requirements> and <https://journals.plos.org/plosone/s/figures#loc-preparing-figures-from-image-files>. When you submit your revised manuscript, please ensure that your figures adhere fully to these guidelines and provide the original underlying images for all blot or gel data reported in your submission. See the following link for instructions on providing the original image data: <https://journals.plos.org/plosone/s/figures#loc-original-images-for-blots-and-gels>.

In your cover letter, please note whether your blot/gel image data are in Supporting Information or posted at a public data repository, provide the repository URL if relevant, and provide specific details as to which raw blot/gel images, if any, are not available. Email us at [plosone@plos.org](mailto:plosone@plos.org) if you have any questions.

**Author’s reply:** Thank you. All original uncropped and unadjusted images of gels used in the manuscript have been included in the Supporting information file. All requirements for gel reporting have been met. Images of phenotypic tests conducted and additional gel images are also included in the file as requested by Reviewer #1. I hope this is acceptable to you.

5. We note that you have included the phrase “data not shown” in your manuscript. Unfortunately, this does not meet our data sharing requirements. PLOS does not permit references to inaccessible data. We require that authors provide all relevant data within the paper, Supporting Information files, or in an acceptable, public repository. Please add a citation to support this phrase or upload the data that corresponds with these findings to a stable repository (such as Figshare or Dryad) and provide and URLs, DOIs, or accession numbers that may be used to access these data. Or, if the data are not a core part of the research being presented in your study, we ask that you remove the phrase that refers to these data.

**Author’s reply:** Thank you so much. The issue has been corrected accordingly.

6. Your ethics statement should only appear in the Methods section of your manuscript. If your ethics statement is written in any section besides the Methods, please move it to the Methods section and delete it from any other section. Please ensure that your ethics statement is included in your manuscript, as the ethics statement entered into the online submission form will not be published alongside your manuscript.

**Author’s reply:** Thank you so much. The ethics statement has been moved to the Methods section of the manuscript. I trust this is okay now.

**Review Comments to the Author**

Please use the space provided to explain your answers to the questions above. You may also include additional comments for the author, including concerns about dual publication, research ethics, or publication ethics. (Please upload your review as an attachment if it exceeds 20,000 characters)

**Reviewer #1:**

1-the language needs revise by profisionals

**Author’s reply:** Thank you for the comment. The language of the manuscript has been revised by an academician who is currently a faculty member at University of Kansas Medical Center and has over 100 publications in peer reviewed journals. We trust this is okay now.

2- please add at least 4 ref from middleeast

**Author’s reply:** Thank you for the suggestion. A study from the Middle East was already referenced in this manuscript, and three additional references of studies obtained from the Middle East have been added that discuss data similar/supportive of the present study. This includes references 30, 43,44 and 45. I hope this is acceptable to you now.

3- add statics to your study

**Author’s Reply:** Thank you for the suggestion. Statistical analysis of significant parts of data has been added to the result section (Line 160 onwards). I hope this is acceptable to you now.

4- whhre is the anaylsis of sequencing??

**Author’s Reply:** Thank you. As per your comment, the analysis of sequence data has been reported through **Fig 1** now, which depicts mutational alterations in both genes studied. I trust this is acceptable:

5- where is figurs of your study?

**Author’s Reply:** Thank you for your question. Figures relevant to this study have been uploaded separately, as per the instructions for figure submission by the journal. I hope this is in accordance to you.

6- please add figure of your phnotypic test

**Author’s Reply:** Thank you. The figures of phenotypic tests relevant to this study have now been uploaded as supporting information. I believe you can find the figures in Supporting Information File titled S1_raw_images, and trust this is acceptable to you.

**Reviewer #2:**

**Major comments:**

- In table 3: You have to create another group without detected mutations mention the number of isolates at each MIC in table 3.

**Author’s reply:** Thank you so much for your suggestion. Quinolone sensitive isolates with MIC ≤4 µg/ml were not subjected to sequence analysis, hence were not included in the table previously. We have now updated Table 3 as per your suggestion, and I hope this is acceptable to you. The updated table is attached below:

**Table 3.** **Association of mutations in QRDR region and tolerance level against fluoroquinolones**

| Group | No. of Isolates | Fluoroquinolone antibiotics | No. of isolates with corresponding MIC (µg/ml) value | | | | | | | | | | |
| --- | --- | --- | --- | --- | --- | --- | --- | --- | --- | --- | --- | --- | --- |
|  |  |  | 0.125 | 0.5 | 1 | 2 | 4 | 8 | 16 | 32 | 64 | 128 |  |
| I; Single alteration in *gyrA*(83) | 12 | Ciprofloxacin |  |  |  |  |  |  | 5 | 5 | 2 |  |  |
|  |  | Levofloxacin |  |  |  |  |  |  | 1 | 6 | 5 |  |  |
| II; Alterations in *gyrA* (83) and *parC* (87) | 3 | Ciprofloxacin |  |  |  |  |  |  |  |  |  | 3 |  |
|  |  | Levofloxacin |  |  |  |  |  |  |  |  |  | 3 |  |
| III; Quinolone sensitive isolates | 38 | Ciprofloxacin | 12 | 25 | 1 |  |  |  |  |  |  |  |  |
|  |  | Levofloxacin |  | 26 | 10 | 1 | 1 |  |  |  |  |  |  |

- Where is the statistical analysis that confirm the significant or non-significant correlation between the detected mutations and the resistance?

**Author’s reply:** Thank you for the question. Statistical analysis of significant parts of data has been added to the result section (Line 160 onwards). Correlation between detected mutations of both *gyrA* and *parC,* and level of tolerance to fluoroquinolones tested has been noted in text as *p*-values. Data as well as statistical analysis confirms the correlation as isolates with double mutation exhibited highest level of tolerance (MIC ≥128 µg/ml). I trust this is acceptable to you now.

Minor comments:

- Please check the language carefully in the whole manuscript e.g. Line101: "were" and Line 36.

**Author’s reply:** Thank you for the suggestion. The grammatical errors have been checked and corrected; I trust this is acceptable now.

- Clarify the abbreviation "QRDR” in line 56, as it the first time to be mentioned in introduction and please clarify other abbreviations.

**Author’s reply:** Thank you for the comment. All abbreviation, including "QRDR” in line 56 has been clarified now. I trust this is okay now.

- In line 61, does the word " motif" refer to the QRDR motif? please clarify.

**Author’s reply:** Thank you. Yes, the word “motif’ refers to the motif of QRDR region of GyrA, more specifically in *P. aeruginosa,* the 67-106^th^ position of the GyrA subunit of DNA Gyrase serves as the QRDR region. Occurrence of mutation at this position leads to structural alteration of DNA Gyrase, changing the motif. This can result in reduced binding affinity of fluoroquinolones to DNA Gyrase, developing resistance. But to make it clearer, I have rephrased the sentence. I hope that clarifies the topic.

- In methods, please mention the ethical approval details.

**Author’s reply:** Thank you so much. The ethics statement was previously added at the end of the manuscript, and has been moved to the Methods section of the manuscript. I hope this is okay.

- Please carefully revise the whole manuscript as it contains some" review track changes" e,g line 78 ??

**Author’s reply:** Thank you. The manuscript has been revised as per your suggestion.

- Please mention the year of the CLSI version that was followed in this study in the references list.

**Author’s reply**: Thank you. In this study, the 31^st^ edition of CLSI published in 2021 was followed. This has been mentioned in the references list [21] and in the methods section as well. I trust this is acceptable to you.

- In figure 1: Indicate the molecular size of different bands of the marker and also indicate its catalogue number

**Author’s reply:** Thank you for the suggestion. The molecular size of different bands as well as the amplified product is now indicated in the figure. Catalogue number of molecular markers used in this study is mentioned in the figure caption. I hope this is acceptable to you. The new figure with caption is:

**Fig 1. PCR based detection of PMQR variants**. Variants *qnr* gene sequences were PCR amplified using specific primers (Table 1). PCR products were electrophoresed through 1% agarose gels, stained with ethidium bromide, and visualized with UV light. The 100 bp DNA ladder (Promega, USA; Catalogue number G2101) indicates the size of PCR products. Lane 2, 6 and 10 represent variant-specific positive controls, and lane 3, 7 and 11 represent negative controls. No isolates were positive in *qnr*A, *qnr*B and *qnr*S specific PCR.

- In table 1:
• Write the name of genes correctly "Italic and small" in column 3 and in the whole manuscript.
• Put the "°C" only in the heading.

**Author’s reply:** Thank you for the suggestion. The table has been updated and I hope you’ll find it acceptable. The revised table is attached below:

**Table 1. Primers used in this study**

| Gene | Primer Sequence (5’ to 3’) | Use | Annealing Temperature (°C) | Amplicon Size/ Nucleotide Position | Reference |
| --- | --- | --- | --- | --- | --- |
| *gyrA* | GAC GGC CTG AAG CCG GTG CAC | Amplification and sequencing *of* *gyrA* subunit of Toposiomerase II | 65 | 115-135 | [22] |
|  | GCC CAC GGC GAT ACC GCT GGA |  |  | 531-511 |  |
| *parC* | CGA GCA GGC CTA TCT GAA CTA T | Amplification and sequencing of *parC* subunit of Toposiomerase IV | 55 | 63-84 |  |
|  | GAA GGA CTT GGG ATC GTC CGG A |  |  | 366-344 |  |
| *qnrA* | AGA GGA TTT CTC ACG CCA GG | Amplification of *qnrA* | 54 | 580bp | [23] |
|  | TGC CAG GCA CAG ATC TTG AC |  |  |  |  |
| *qnrB* | CCT GAG CGG CAC TGA ATT TAT | Amplification of *qnrB* | 60 | 390bp | [24] |
|  | GTT TGC TGC TCG CCA GTC GA |  |  |  |  |
| *qnrS* | GCA AGT TCA TTG AAC AGG GT | Amplification of *qnrS* | 54 | 428bp | [23] |
|  | TCT AAA CCG TCG AGT TCG GCG |  |  |  |  |
| fd1 | TCT AAA CCG TCG AGT TCG GCG | Amplification and sequencing of 16S rDNA | 42 | 1500bp | [25] |
| rp2 | ACG GCT ACC TTG TTA CGA CTT |  |  |  |  |

- - Write the name of protein correctly "Not Italic and start with capital letter" in Table 2 and in the whole manuscript when it refers to protein but not gene.

**Author’s reply:** Thank you for your comment. The names have been corrected accordingly and I trust this is acceptable.
