# Supplementary material for: Mutational alterations in the QRDR regions associated with fluoroquinolone resistance in Pseudomonas aeruginosa of clinical origin from Savar, Dhaka
Source: PLoS One. 2025 Feb 24;20(2):e0302352. doi: 10.1371/journal.pone.0302352 (PMC12002805; doi:10.1371/journal.pone.0302352)
Supplement: Attachment — Submitted filename: Response to Reviewers R1.docx [file pone.0302352.s005.docx]

**Mutational alterations in the QRDR regions associated with fluoroquinolone resistance in Pseudomonas aeruginosa of clinical origin from Savar, Dhaka**

**Review Comments to the Author:**

Reviewer #3: This paper examines 53 clinical isolates of Pseudomonas aeruginosa (PA) for fluoroquinolone resistance and associated resistance mechanisms, including mutations in the GyrA and ParC genes as well as the presence of plasmid-mediated quinolone resistance (PMQR) genes.

- The research question and specific knowledge gaps are not clearly defined.

**Author’s Reply:** Thank you for the comment. Detailed information focusing on research question is now included in the Introduction section of the manuscript. We believe this is acceptable now.

- Additionally, the detection of GyrA mutations in P. aeruginosa isolates from Bangladesh is not a new finding, hence, its contribution to the existing body of knowledge is limited. Moreover, the regional focus of the study limits the broader generalizability of its findings.

**Author’s Reply:** Thank you for the question. While we agree that GyrA mutations in *P. aeruginosa* is not a new finding, we also believe that surveillance of antimicrobial resistance and resistance determinants are crucial to understand trends in AMR. The present study also focuses on the dissemination of PMQR genes in high-complexity hospitals, as they can serve as a reservoir for mobile genetic elements. We believe regional data is an integral part of global AMR surveillance, and contributions like our study helps to understand recent trends in AMR globally.

- Although the sources of the isolates were broadly mentioned, the specific distribution of isolates across different clinical sample types was not provided, which could be important for identifying high-risk sample categories. Additionally, the procedures for the primary isolation of the organism were not described. Including population-related data, such as the number of patients with specific complications tested for the organism, would help estimate the prevalence of fluoroquinolone-resistant Pseudomonas aeruginosa.

**Author’s Reply:** Thank you for the comment. Data regarding distribution of isolates and population related data have been previously published in an article that described the source of the isolates used in this study (Anjum *et al,* 2023). Hence the isolates are labeled as “Laboratory Collection” in the present manuscript. We have refrained from including the same data in this manuscript, as well as the procedures of primary isolation and population related data, to avoid repetition and redundancy. However, based on your suggestion, we will be attaching the sample distribution and population related data as supplementary data. We believe this is okay and hope is acceptable to you.

- Furthermore, it is unclear why the study focuses exclusively on fluoroquinolone resistance, without addressing resistance to other antibiotics, which would provide a more comprehensive understanding of the resistance profile.

**Author’s Reply:** Thank you for your question. The present investigation is part of an ongoing project that aims to investigate the multidrug resistance in clinical infection caused by different pathogenic bacteria, including *P. aeruginosa* from Savar, Bangladesh*.* It also studies the resistance mechanisms to different groups of clinically important antibiotics including β-lactams, carbapenems and fluoroquinolones. Since the data obtained is large in quantity and would be easy to describe separately, we chose to prepare manuscripts focusing different antibiotics. Our previous studies have already discussed the resistance determinants to β-lactams (Anjum *et al,* 2023) and carbapenems among the *P. aeruginosa* isolates used in this study. The mentioned article addresses the MDR nature of the isolates and discusses their AMR profiles. The current manuscript, therefore, is solely focused to showcase data regarding resistance to fluoroquinolones in *P. aeruginosa* from Savar, Dhaka. We hope this is acceptable to you.

Specific comments:
• Please provide a brief description of the reconfirmation of isolates using standard culture methods.

**Author’s Reply:** Thanks for the suggestion. A brief description of standard culture method for identification of *P. aeruginosa* is now included in the Materials and Methods section of the manuscript. We believe this is okay now.

• Although it was not mentioned but I assumed P. aeruginosa ATCC 27853 was used as a positive control. Did you use any negative control? If yes, please mention that too.

**Author’s Reply:** Thank you for your question. Yes, *P. aeruginosa* ATCC 27853 was used as positive control. And as negative control, *E. coli* ATCC 25922 was used in this study. This information is now mentioned in the text. We trust this is okay now.

• How did you purify the PCR product. Please describe briefly.

**Author’s Reply:** Thank you for the question. PCR products were purified using Wizard® SV Gel and PCR Clean-Up System from Promega, USA (Catalog Number: A9281). The clean-up system is now mentioned in the text. We believe this is acceptable now.

• Why did you not test GyrB and ParE genes? Provide a reasonable justification.

**Author’s Reply:** Thank you for your question. Although our initial plan was to investigate mutational alterations in the relevant subunits of both Topoisomerase II (GyrA and GyrB) and Topoisomerase IV (ParC and ParE), the study was limited financially. Since we had limited funding, we chose to focus on the major subunits of both enzymes (GyrA and ParC). These subunits are more prone to alterations and are more frequently associated with fluoroquinolone resistance, so we chose to investigate these first. We are hoping to expand the present study by investigating GyrB and ParE alongside other mutational alterations associated with multidrug resistance like *nfxC, nfxB* and *mexR* once further fundings are available. We hope this explains your query.

• Which control strains did you use in tests for QRDR genes?

**Author’s Reply:** Thank you for the question. For PCR and sequence analysis of QRDR genes, *P. aeruginosa* PAO1 was used as a control strain. Obtained sequences were compared with associated *P. aeruginosa* PAO1 loci from NCBI GenBank as reference. We hope this answers your question.

• The background section mentions other mechanisms of resistance; however, these were not investigated in the study. This creates gaps in understanding the complete resistance mechanisms among the isolates.

**Author’s Reply:** Thank you for the comment. In addition to mutational alterations in QRDR regions and acquisition of PMQR genes, fluoroquinolone resistance can be mediated by overexpression of efflux pumps like MexAB-OprM. We have also explored the phenotypic overexpression of efflux pump in efflux inhibitor induced MIC depression assay, but didn’t find any significant efflux pump activity. This has been discussed in the discussion section (Line 214 onwards). We hope this is acceptable to you.

• Please replace the term “tolerance level” with “MIC value” throughout the manuscript.

**Author’s Reply:** Thank you for the suggestion. We have replaced “tolerance level” with “MIC value” throughout the manuscript in relevant contexts. We trust this is okay now.

• What was the rationale for testing only ciprofloxacin and levofloxacin? Why were other antibiotics within the same class not included in the analysis?

**Author’s Reply:** Thank you for the question. Fluoroquinolones like ciprofloxacin and levofloxacin are among the most commonly prescribed and therapeutically used antibiotics of the class. Ciprofloxacin is a second-generation fluoroquinolone, while levofloxacin belongs from the third generation of the class. These antibiotics are the most frequently prescribed fluoroquinolones at the hospitals the isolates are obtained from, and that is the main rationale for selecting these antibiotics for the current study. We hope this answers your question.

• Please provide the GenBank accession numbers for the sequences of all isolates.

**Author’s Reply:** Thank you for the suggestion. The present study investigated the alterations of *gyrA* and *parC* subunits for mutational alterations. Since all of the isolates had similar alterations in their *gyrA* and no novel mutations were identified from the sequence obtained, we decided to submit the sequence from a representative isolate. Similarly, the *parC* sequence obtained from a representative isolate is submitted to the GenBank. Their accession numbers PP501828 *(gyrA)* and PP526740 *(parC)* are already mentioned in this study. Sequences of all isolates are not submitted to the GenBank to avoid repetition/ redundancy, but are now available as supplementary data. We hope this is acceptable to you.

• Which positive and negative controls were used in the sequencing analysis? Please also provide details of the control strains used in the PCR for detecting PMQR genes.

**Author’s Reply:** Thank you for the question. For sequence analysis, *P. aeruginosa* PAO1 was used as a positive control and obtained sequences were compared with associated *P. aeruginosa* PAO1 loci from NCBI GenBank as reference. In PCR for PMQR gene detection, locally curated control strains *E. coli* DMC1 (for *qnrA* and *qnrB*) and *E. coli* Shishu3 (for *qnrS*) was used as positive control. These control strains were gift from icddr.b (International Centre for Diarrheal Disease Research, Bangladesh), and have been confirmed to harbor the mentioned PMQR genes. The controls are now mentioned in the manuscript. We believe this is okay now.

• Including a figure displaying the amino acid sequence alignment (multiple alignment) of the 15 isolates, particularly highlighting the regions with alterations, would improve the clarity of the findings.

**Author’s Reply:** Thank you for the proposition. We believe we have already included two figures displaying the alignment of amino acid sequences highlighting regions with alterations in the manuscript (Fig 1: Sequence analysis of gyrA and parC showing alterations). We trust that is okay.

• What are the current treatment guidelines for Pseudomonas aeruginosa infections? It would be valuable to frame the discussion around the clinical relevance of your study, particularly concerning fluoroquinolone resistance. If fluoroquinolones are not the drugs of choice, what are the implications of your findings?

**Author’s Reply:** Thank you for the suggestion. We have included a section in the Discussion part of the manuscript explaining the importance of fluoroquinolones from a therapeutic point-of-view, and why it is relevant to study fluoroquinolone resistance in *P. aeruginosa*. We trust this is acceptable now.

Reviewer #4:

1- the authors used statics in the paper but didn't mention the paragraph belong to it.

**Author’s Reply:** Thank you for the suggestion. A subsection titled “Statistical Analysis” is now included in the Materials and Methods section of the manuscript. We trust this is okay now.

2- the figure 2 repeated two times.

**Author’s Reply:** Thank you for the enquiry. However, we could not find the repetition mentioned in the manuscript. We believe we have submitted the figure separately and it’s not included in the manuscript file.

3- in figure two the author claim in some lines no results but each lines has some band? this confused results specially found line with negative results.

**Author’s Reply:** Thank you for the question. The authors claim the results in figure two as negative since no specific amplification is observed in the mentioned lanes. Amplification often produces non-specific amplicons that can be observed as faint bands in places not corresponding to the actual amplicons. Hence, positive controls are used and non-specific amplifications are considered as negative result. We hope this clears the confusion and is acceptable now.

4- the paper didn't has study limitation

**Author’s Reply:** Thank you for the comment. Limitations of the present study is now discussed briefly in the Discussion section of the manuscript. We hope this is acceptable to you.
